# A Deforming Fibrous Band From an Os Intermetatarseum as a Rare Cause of Rigid Adolescent Hallux Valgus: A Case Report

**DOI:** 10.7759/cureus.92422

**Published:** 2025-09-16

**Authors:** Ahmet T Yildirim, Akshay R Reddy, Caitlin Curtis Crocker, Delaney Lagrew, Miqi Wang

**Affiliations:** 1 Department of Orthopedic Surgery and Sports Medicine, University of Florida College of Medicine, Gainesville, USA; 2 College of Medicine, University of Florida, Gainesville, USA

**Keywords:** accessory ossicle, adolescent hallux valgus, akin osteotomy, distal chevron osteotomy, fibrous band, hallux valgus surgery, os intermetatarseum

## Abstract

Hallux valgus (HV) is a common forefoot deformity, and an associated os intermetatarseum is often an incidental and asymptomatic radiographic finding. The role of an os intermetatarseum as a direct etiological factor in HV, particularly when mediated by an anomalous structural tether causing deformity, is unusual.

We describe the case of a 16-year-old female who presented with several years of progressive bilateral foot deformity and pain. Clinical examination revealed a rigid moderate HV deformity on the right and a flexible mild HV on the left. Plain radiographs confirmed bilateral HV and an os intermetatarseum fused to the base of each second metatarsal. Advanced imaging with CT and MRI of the right foot further identified an anomalous fibrous band extending from the os intermetatarseum to the fibular sesamoid and lateral base of the proximal phalanx of the hallux. The patient underwent surgical correction, including the excision of the os intermetatarseum and associated anomalous fibrous band, a first metatarsal Chevron osteotomy, and a proximal phalanx Akin osteotomy. Intraoperatively, this band was confirmed to exert an adduction force on the hallux. At the 12-week postoperative follow-up, the patient reported significant pain relief. Radiographs showed stable alignment with healed osteotomies, and she was cleared for a gradual return to sports.

An os intermetatarseum, when associated with an anomalous fibrous band, can act as a direct cause of HV deformity that must be addressed separately if surgical intervention is indicated. Clinicians should consider this rare anatomical variant in the diagnostic workup of unusual or rigid HV presentations, particularly in younger patients or those with a history of other congenital foot anomalies. CT and MRI can be instrumental in identifying such structures preoperatively, allowing for targeted surgical intervention to address all contributing factors for an optimal outcome.

## Introduction

The os intermetatarseum is a rare accessory ossicle commonly found between the bases of the first and second metatarsals and/or projecting from the medial cuneiform [1]. The ossicle may be independent, fused to form an exostosis within the intermetatarsal space, or articulating with a joint. The os intermetatarseum is much less common than the os trigonum and the os peroneum, with an estimated prevalence ranging from 1.2 to 10% [1,2]. Patients are frequently asymptomatic, with the os intermetatarseum often being found incidentally. Characteristics of the ossicle vary in size and shape across patients. However, cases in which patients present with dorsal foot pain and paresthesias due to compression of the peroneal nerves have been reported [3-6]. The os intermetatarseum has also been suggested to be associated with hallux valgus (HV). HV is a common forefoot deformity, affecting an estimated 23% of adults aged 18-65 years [7-9]. The prevalence of juvenile HV was estimated at a global rate of 11% in those under 20 by a recent meta-analysis [10], whereas a large European cohort study identified a prevalence of 3.5% in adolescents aged 13-18 [11]. Reports of patients with both HV and os intermetatarseum are rare, and the association is often attributed to the os intermetatarseum forcing the first metatarsal into a varus position [5,7,12,13]. However, a direct deforming mechanism involving an anomalous fibrous band tethering the hallux is exceptionally rare. The band's tethering force on the proximal phalanx can directly contribute to the HV deformity. This specific pathology appears to have been clearly described only once previously in the literature by Henderson et al. in the 1960s, demonstrating the rarity of this finding.

This report presents the case of an adolescent patient with HV, where this unique pathology was addressed. Her surgical management of the right foot included a Chevron osteotomy, an Akin osteotomy, and debridement of the ossicle and the anomalous band. In reporting this case, we aim to influence future surgical approaches and decision-making when treating HV caused by the rare combination of an os intermetatarseum and a deforming fibrous band.

## Case presentation

A 16-year-old female with a history of right foot sixth digit amputation over a decade prior for congenital postaxial polydactyly presented in March 2023 with bilateral foot pain and deformity. The pain started a few years ago and progressively worsened. Pain was localized primarily to the medial aspects of both hallux metatarsophalangeal (MTP) joints (rated 8/10 when exacerbated). Initially, nonoperative treatment with wide-toebox shoes offered inadequate relief. The patient then developed new-onset foot numbness, more pronounced on the right. Physical examination revealed painful medial eminences at both hallux MTP joints with mild contractures of lesser toes. Tenderness over the first MTP joint bilaterally was present. The right HV was not passively correctable, while the left was correctable. There was no pain with the range of motion. The grind test was negative bilaterally, suggesting an absence of advanced arthritis at the first MTP joints [14]. Ankle dorsiflexion was 10 degrees with the knees extended bilaterally, and protective plantar sensation was intact.

**Figure 1:**
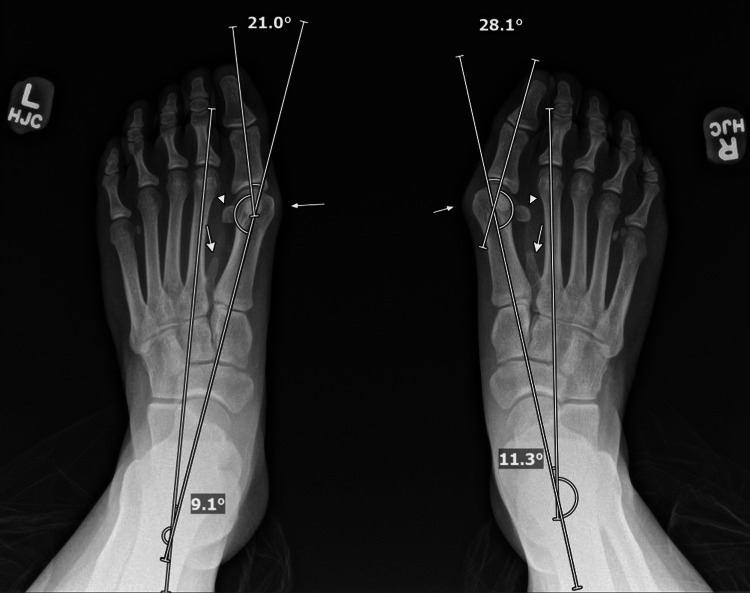
Preoperative anteroposterior (AP) weight-bearing radiograph of both feet. The radiograph shows the following measurements: Right foot: intermetatarsal angle (IMA) of 11.3°, hallux valgus angle (HVA) of 28.1 °; left foot: IMA of 9.1°, HVA of 21.0°. Arrows indicate the prominent medial eminence and the os intermetatarseum bilaterally. Triangles indicate the laterally displaced fibular sesamoids bilaterally.

Initial weight-bearing radiographs demonstrated bilateral os intermetatarseum fused to the base of the second metatarsal and moderate HV on the right and mild HV on the left (Figure 1). HV angle (HVA) was 28.1° and intermetatarsal angle (IMA) 11.3° for the right foot and 21.0° and 9.1° for the left foot, respectively. Bilateral sesamoid rotation was noted. There was no significant MTP joint space narrowing.

**Figure 2:**
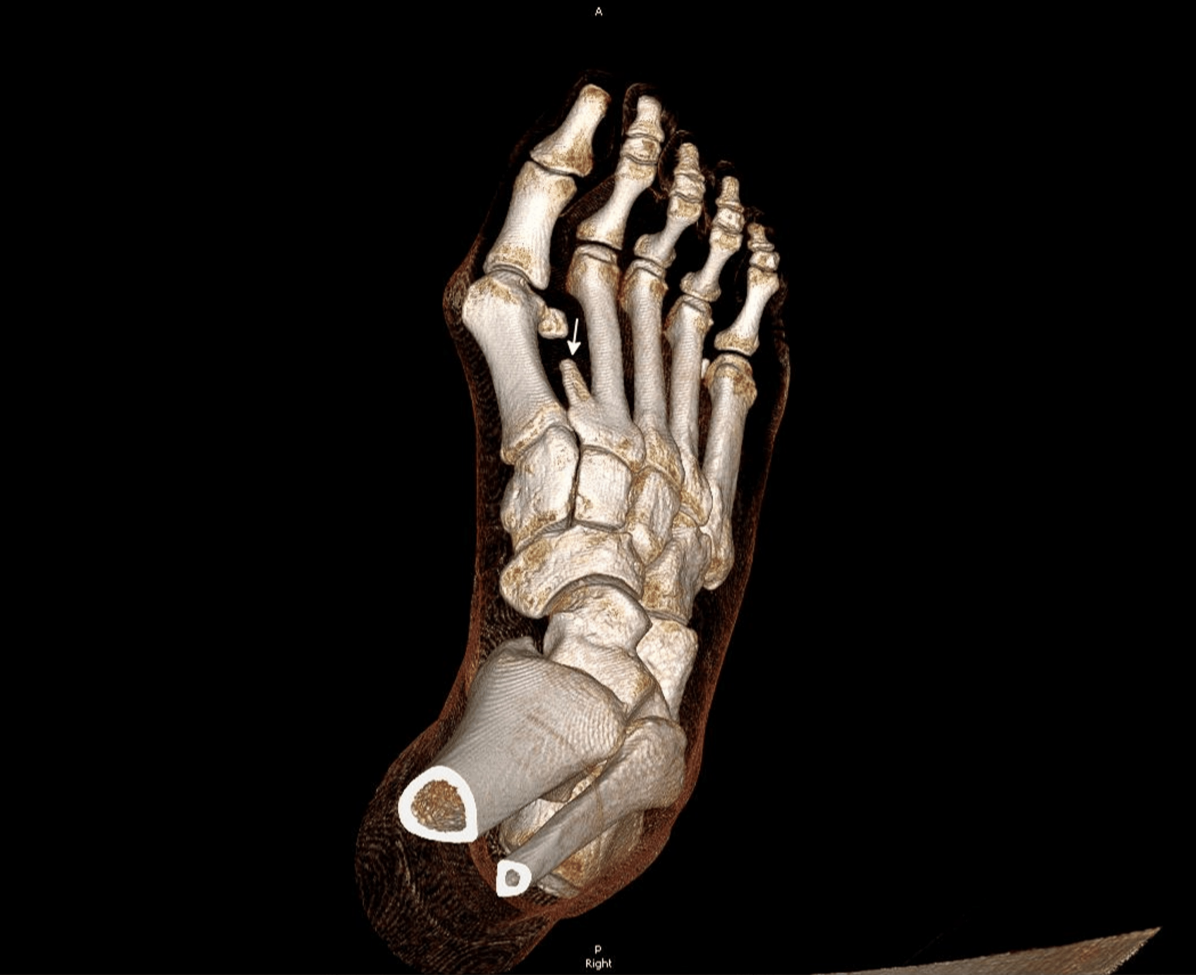
Preoperative 3D CT reconstruction of the right foot, anteroposterior (AP) view. The image details the os intermetatarseum (arrow) fused to the medial base of the second metatarsal, its distinct morphology, and its spatial relationship with the first metatarsal.

**Figure 3:**
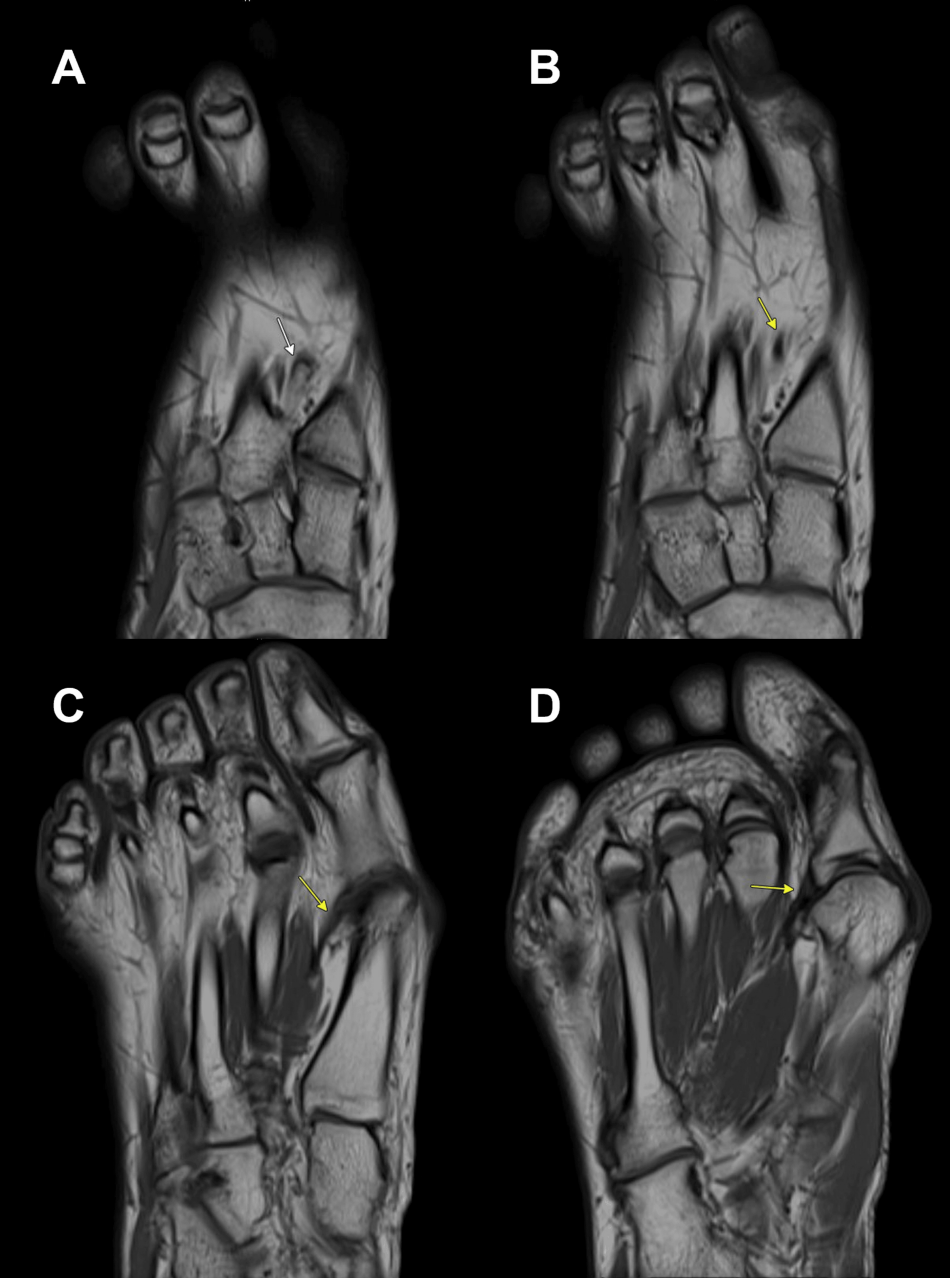
Preoperative axial T1-weighted MRI of the right foot showing the os intermetatarseum and the associated anomalous fibrous band. A: the os intermetatarseum (white arrow) is visualized, fused to the medial base of the second metatarsal; B-D: the T1-hypointense anomalous fibrous band (yellow arrow) is visualized originating from the os intermetatarseum and extending distally.

**Figure 4:**
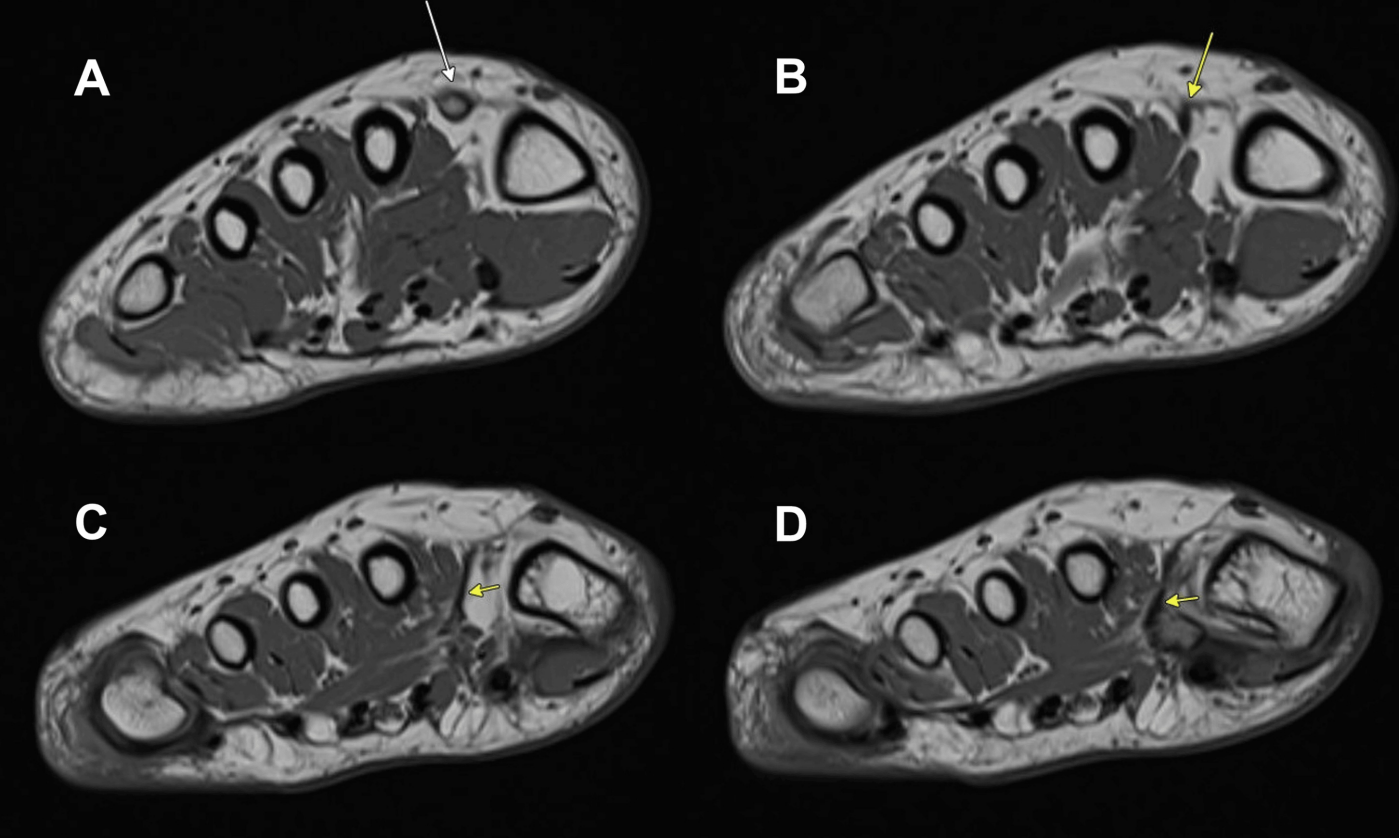
Preoperative coronal T1-weighted MRI of the right foot, demonstrating the os intermetatarseum and the associated anomalous fibrous band. A: the os intermetatarseum (white arrow); B-D: the T1-hypointense anomalous fibrous band (yellow arrow) is visualized originating from the os intermetatarseum and extending distally.

CT and MRI of the right foot confirmed HV with lateral sesamoid subluxation. The CT delineated the fused os intermetatarseum arising from the medial base of the second metatarsal, directed distally (Figure 2). Crucially, MRI identified a linear, T1/T2 hypointense fibrous band arising from the tip of this os, appearing contiguous with the fascia of the first dorsal interosseous muscle and extending to the lateral base of the first proximal phalanx of the hallux and fibular sesamoid (Figures 3, 4). This anomalous band was suspected to be a deforming factor and the cause of the rigidity of the deformity.

In mid-April 2023, these imaging results were reviewed with the patient. The anomalous band was highlighted as the likely cause of the right foot's rigidity. A decision was made for surgical intervention on the right foot, to include a Chevron osteotomy, an Akin osteotomy, and excision of the os intermetatarseum remnant with the associated anomalous band. Informed consent was obtained. She expressed a goal to play basketball by August.

Operative technique

**Figure 5:**
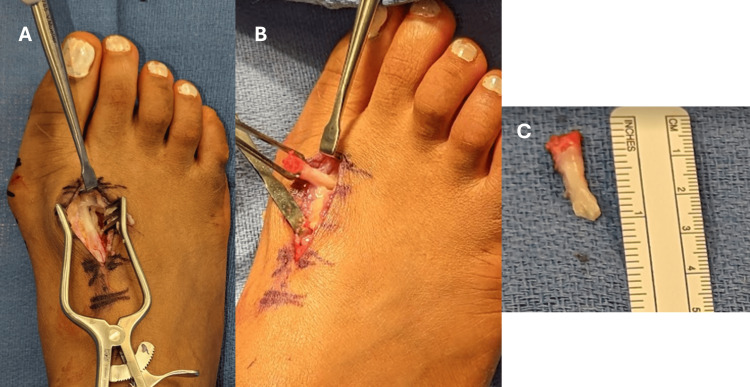
Intraoperative photographs of the anomalous fibrous band and os intermetatarseum. A: in situ view of the tense fibrous band attached to the os intermetatarseum; B: the band after its release; C: the excised os intermetatarseum with its attached fibrous band.

In late May 2023, the patient underwent the planned operative procedures on her right foot. After administration of general anesthesia supplemented with a regional block and routine preoperative timeouts and antibiotic administration, she was placed in the supine position with all bony prominences well-padded. The procedure began with addressing the os intermetatarseum. A 3 cm longitudinal incision was made over the palpable ossicle in the first intermetatarsal space. Dissection was carried down carefully to the bone, protecting neurovascular structures. The os intermetatarseum and an associated thick, anomalous soft tissue tether were identified. The tissue was 5-7 mm in diameter and tendon-like in appearance. Traction upon the tissue caused increased adduction of the great toe, and varus angulation of the first MTP caused the tissue to tighten. The tether and a portion of the os intermetatarseum totaling 2 cm were resected, which led to increased passive mobility of the HV deformity (Figure 5). The resected ossicle and tether were sent for pathological examination.

**Figure 6:**
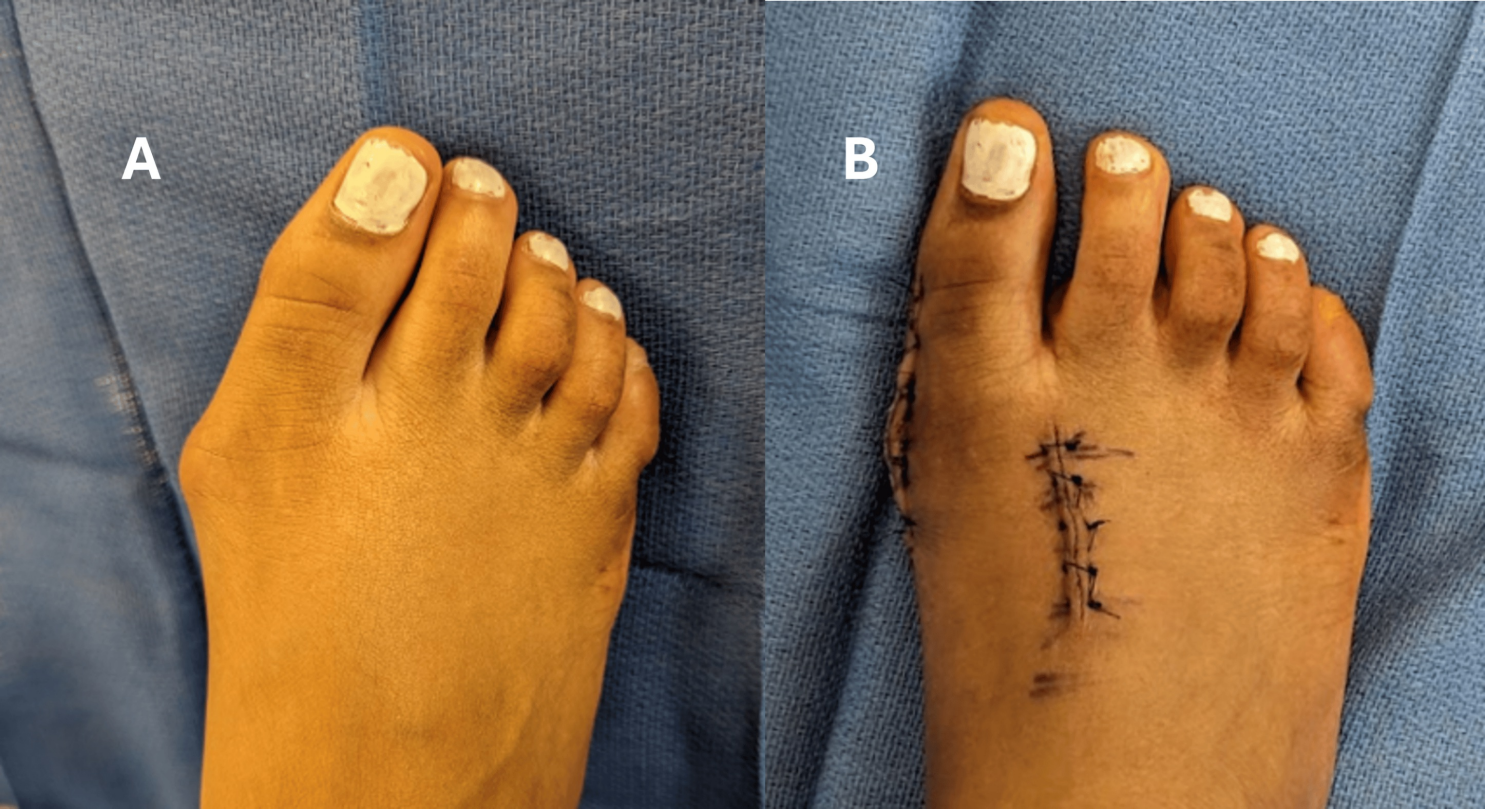
Intraoperative clinical photographs of the right foot. A: intraoperative view prior to surgical correction, showing the hallux valgus; B: intraoperative view after surgical correction, demonstrating the improved alignment of the great toe and reduction of the medial prominence.

Next, the HV correction was performed. A silver osteotomy was performed through a medial first MTP incision. The lateral soft tissues were piecrusted. A 60-degree Chevron osteotomy [15] was then created in the first metatarsal head. The distal capital fragment was translated laterally to correct the IMA, and this correction was stabilized with two 3.0 mm x 18 mm headed screws inserted from dorsal to plantar. Excess bone from the step-off was carefully removed, and any sharp edges were smoothed. As some residual valgus angulation of the hallux was noted at the proximal phalanx, an Akin osteotomy [16] was performed. A 2 mm medially based closing wedge osteotomy was created at the diametaphyseal junction of the proximal phalanx. This osteotomy was then closed and secured with a single 3.0 mm x 26 mm headed screw, while the bone was held in a reduced position. The medial soft tissues were reefed to improve the reduction of HV (Figure 6).

Pathological results described the resected specimen as lamellar bone and benign fibroadipose tissue, consistent with the os intermetatarseum and the tethered fibrous band.

Postoperative course

**Figure 7:**
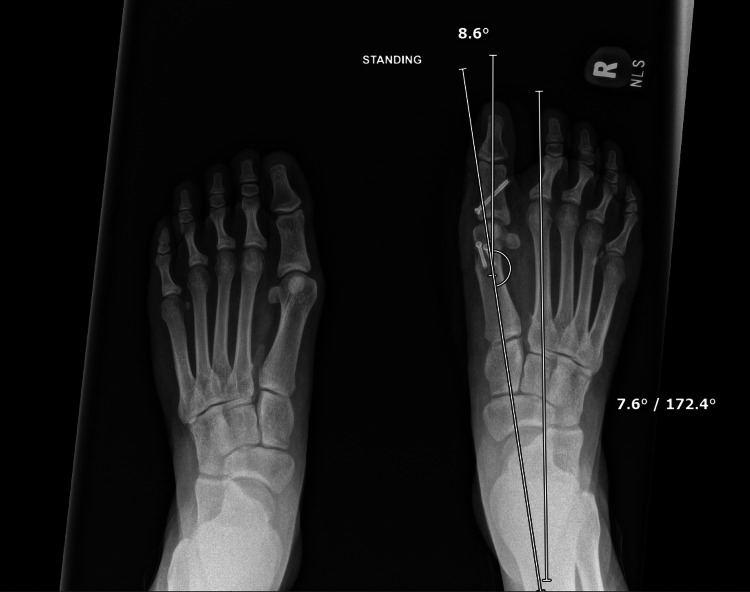
Postoperative anteroposterior (AP) weight-bearing radiograph of both feet at two weeks. Measurements of the right foot are an intermetatarsal angle (IMA) of 7.6° and a hallux valgus angle (HVA) of 8.6°. The overall alignment appears appropriate, and the implants are well-positioned.

The patient had an uneventful early recovery from her right foot surgery. Surgical sutures were removed approximately two weeks postoperatively in mid-June 2023. Her incisions were healing well, and the physical exam was reassuring. She was instructed on gentle range-of-motion exercises and continuing protected ambulation in a postoperative shoe. Radiographs at this visit confirmed well-positioned implants, appropriate alignment, and a visibly shortened os intermetatarseum with early healing. HVA and IMA were 8.6° and 7.6°, respectively (Figure 7).

At six weeks postoperatively, the patient had discontinued use of the postoperative shoe and returned to closed-toe sneakers. As such, she had some recurrence of the HV deformity but had no pain with shoewear. At 12 weeks postoperatively, the patient had no pain or stiffness with ambulation in regular shoes. No additional change in HVA was noted. She then returned to all normal activities gradually, including basketball.

## Discussion

This report details the successful short-term surgical management of symptomatic HV in an adolescent with a rare underlying cause: an os intermetatarseum associated with an anomalous fibrous band acting as a direct deforming tether. Preoperative identification via advanced imaging and intraoperative confirmation of this band were crucial. Surgical treatment, combining targeted excision of the band and os intermetatarseum remnant with corrective osteotomies, led to significant pain relief, stable alignment, and return to sport after 12 weeks. This case highlights the importance of further evaluation for atypical HV presentations. While weight-bearing radiographs are generally sufficient for typical, flexible deformities, atypical findings such as a rigid HV in an adolescent without arthritis, as seen in our patient, warrant further investigation [17].

The defining feature of our case was the anomalous fibrous band connecting the os intermetatarseum to the hallux, which acted as a direct deforming mechanism for HV. Henderson (1963) [12] provided a key historical account of a similar ‘tendinous structure’ originating from an os intermetatarseum and inserting on the lateral proximal phalanx of the hallux. He identified this finding in siblings and histologically confirmed it as tendon tissue. He posited this might be a “lost first plantar interosseous muscle.” He noted resection of the band, even without first metatarsal osteotomy in one case, yielded excellent deformity correction. The profound impact of this band in our case was similarly confirmed intraoperatively. The band's attachment to the fibular sesamoid is a particularly critical finding. Lateral displacement and rotation of the sesamoid are key components of the three-dimensional HV deformity. By directly tethering the fibular sesamoid, this anomalous band in our patient inhibited the correction of this malalignment and worsened the three-dimensional deformity. In contrast, other reports of os intermetatarseum with HV, where no such band was detailed, did not have correction of HV with os intermetatarseum excision alone [5]. In addition, Henderson [12] hypothesized that the band was a vestigial structure. Our patient had a history of postaxial polydactyly, which could support the idea that the resected band was of a congenital origin. The precise embryology and incidence of disease warrant further research, given the limited literature available on os intermetatarseum development, polydactyly, and specific anomalous bands.

The decision to proceed with advanced imaging was driven by the key clinical finding of a rigid HV in a 16-year-old patient who had no radiographic evidence of arthritis or other bony deformities. As rigidity is atypical in an adolescent, an underlying soft tissue mechanical block was suspected. While CT was valuable for detailing the os intermetatarseum itself, magnetic resonance imaging was essential for soft tissue evaluation. In line with literature supporting the selective use of MRI for suspected soft tissue pathology in complex HV cases [18], MRI proved indispensable. It successfully identified the anomalous fibrous band, confirming the clinical suspicion of a tether and critically guiding the surgical plan to include its excision.

The surgical strategy for this adolescent involved a comprehensive approach. This included not only established HV corrective osteotomies (Chevron and Akin) [15,16,19,20] but also the critical excision of the deforming band and os intermetatarseum remnant. Omitting the latter, as suggested by Henderson’s experience, would likely have compromised the outcome. The HVA was corrected to 8.6°, and the IMA to 7.6°, both values well within normal radiographic limits (HVA <15°, IMA <9°) [21]. The patient achieved significant functional improvement by 12 weeks and returned to sports. The robust healing potential in adolescents [22] likely contributed to the osteotomy union and stable result at final follow-up.

The primary limitation is the 12-week follow-up, precluding assessment of long-term deformity stability.

## Conclusions

This case demonstrates that an os intermetatarseum with an associated anomalous fibrous band may be a surgically correctable cause of adolescent HV. It underscores the need for a high index of suspicion for underlying anatomical variants in atypical, rigid HV presentations, especially in younger patients or those with other congenital foot anomalies. Advanced imaging, including MRI for soft tissue detail and CT for osseous anatomy, is key for an accurate diagnosis in these atypical presentations. Surgical plans must be tailored to address all contributing pathologies to optimize outcomes.
